# Portable Quartz Crystal Resonator Sensor for Characterising the Gelation Kinetics and Viscoelastic Properties of Hydrogels

**DOI:** 10.3390/gels8110718

**Published:** 2022-11-07

**Authors:** Andrés Miranda-Martínez, Hongji Yan, Valentin Silveira, José Javier Serrano-Olmedo, Thomas Crouzier

**Affiliations:** 1 Centre for Biomedical Technology (CTB), Universidad Politécnica de Madrid (UPM), 28040 Madrid, Spain; 2 Division of Glycoscience, Department of Chemistry, School of Engineering Sciences in Chemistry, Biotechnology and Health, KTH, Royal Institute of Technology, 106 91 Stockholm, Sweden; 3 AIMES—Center for the Advancement of Integrated Medical and Engineering Sciences, Karolinska Institutet and KTH—Royal Institute of Technology, 114 28 Stockholm, Sweden; 4 Department of Neuroscience, Karolinska Institutet, 171 77 Stockholm, Sweden; 5 Division of Wood Science and Technology, Department of Forest Biomaterials and Technology, SLU, Swedish University of Agricultural Sciences, 756 51 Uppsala, Sweden; 6 Networking Research Center of Bioengineering, Biomaterials and Nanomedicine (CIBER-BBN), Universidad Politécnica de Madrid (UPM), 28040 Madrid, Spain

**Keywords:** covalently crosslinked hydrogels, hydrogel kinetics characterisation, physically crosslinked hydrogels, quartz crystal resonator

## Abstract

Hydrogel biomaterials have found use in various biomedical applications partly due to their biocompatibility and tuneable viscoelastic properties. The ideal rheological properties of hydrogels depend highly on the application and should be considered early in the design process. Rheometry is the most common method to study the viscoelastic properties of hydrogels. However, rheometers occupy much space and are costly instruments. On the other hand, quartz crystal resonators (QCRs) are devices that can be used as low-cost, small, and accurate sensors to measure the viscoelastic properties of fluids. For this reason, we explore the capabilities of a low-cost and compact QCR sensor to sense and characterise the gelation process of hydrogels while using a low sample amount and by sensing two different crosslink reactions: covalent bonds and divalent ions. The gelation of covalently crosslinked mucin hydrogels and physically crosslinked alginate hydrogels could be monitored using the sensor, clearly distinguishing the effect of several parameters affecting the viscoelastic properties of hydrogels, including crosslinking chemistry, polymer concentrations, and crosslinker concentrations. QCR sensors offer an economical and portable alternative method to characterise changes in a hydrogel material’s viscous properties to contribute to this type of material design, thus providing a novel approach.

## 1. Introduction

Hydrogels have found a plethora of applications in biomedicine due to their unique properties such as viscosity, porosity, biocompatibility, and high water content. An example is their use as cell encapsulation materials [1,2,3,4]. Hydrogels are typically formed by polymeric networks of natural or synthetic origin, crosslinked by covalent or noncovalent interactions [2,4,5]. New hydrogel systems are continuously being developed to address their current limitations and expand into new applications. For instance, hydrogel bioactivity and rheology are engineered to be used as ink to form scaffold matrices in 3D bioprinting.

Injectivity depends on the viscous properties of the material, so the proper design of the material viscosity helps in optimising flow in the injector [6,7]. There is also a large body of work on optimising hydrogels in their chemical nature and molecular structures to exhibit immune-modulating properties that can help in treating chronic wounds (or cancer) or promote complex tissue (re)generation processes [8,9]. In the design process of such biomaterials, the material’s mechanical properties are critical. For example, the mechanical properties of implantable hydrogels are often engineered to match those of the surrounding tissue. The material stiffness can also direct the behaviour of cells by, for instance, directing the differentiation of stem cells [10], or the growth kinetics of cancer cells or biopsies [11]. More practically, mechanical properties determine how the gels can be handled and can be used as a marker of biodegradation.

In vivo, the mechanical properties of natural hydrogel materials also define their functions. One example is the mucus gel covering the lungs, gut, and cervix. It depends on their mechanical properties to properly protect epithelial surfaces from shear stresses and pathogen infections, and for their constant recycling and turnover [12,13,14]. In cystic fibrosis patients, the lung mucus is thickened and cannot be removed from the respiratory tract, which leads to infections. Synovial fluid, which is rich in hyaluronic acid, relies on its unique rheological properties to effectively dampen mechanical stresses and lubricate joints [15].

The most common method used for analysing the viscous properties of hydrogels is rheology, which allows for the measurement of gelling kinetics, elastic modulus, viscous modulus, and loss factor [2,16,17,18]. Other important parameters, such as the average molecular weight (Mc, the molecular weight of chain segments between two adjacent crosslinks or entanglement points) and mesh size (ξ, the distance between two adjacent crosslinks or entanglement points), could be further estimated [19]. These measurements provide an accurate and detailed characterisation of hydrogel systems, but require bulky and expensive equipment. In addition, the large variability in protocols applied to study rheological properties complicates comparing the physical properties of hydrogels, and the required volume of the samples is relatively large [18]. There is, thus, a need for standard, affordable, compact methods that all research groups can implement to quickly assess the relative mechanical properties of their hydrogel systems. Such techniques could enable faster interaction in developing new hydrogel systems and could be used to compare hydrogel systems.

Quartz crystal resonators (QCRs) are devices based on the piezoelectric properties of quartz and used as thickness-shear-mode acoustic wave devices [20]. The resonance frequency of a quartz crystal changes with the mechanical load, which results, for instance, from molecules adsorbing to its surface. QCRs are mostly applied to sense mass, such as the quartz crystal microbalance (QCM) [21,22,23]. However, QCRs can also measure fluid viscosity [24,25,26]. One can determine the film’s thickness, shear modulus, and viscoelastic phase angle by measuring the dissipation of the acoustic wave in a film adhered to the quartz crystal’s surface [27]. Given these possibilities and the fact that QCRs require minimal sample volume with which to work, they are good candidates for sensing changes in mechanical properties occurring during the formulation of a polymeric network of hydrogels. Therefore, we propose a new device with its methodology as an alternative to estimate the viscous properties of hydrogels. The resulting analysis could be simpler than that of rheometry; however, it provides a characterisation of the hydrogel formation that aids in the design of the material quickly, simply, and economically.

In this work, we propose a portable and low-cost method for analysing the gelation kinetics and viscous properties of various hydrogels using a sensor based on QCRs. As proof of concept, the gelation of covalently crosslinked mucin hydrogels and physically crosslinked alginate hydrogels was studied. The instrument, weighing less than 100 g and costing less than EUR 200, could consistently measure changes in the viscous properties of the solution, and the measurements were sensitive to key gelling parameters such as polymer concentration and crosslinking chemistry.

## 2. Results

### 2.1. QCR Can Measure the Gelation Kinetics of Covalently Crosslinked Hydrogels

We first investigated whether the QCR sensor could sense the gelation kinetics of a hydrogel system composed of two components formed via covalent bonds. Our group previously developed such hydrogel systems on the basis of the mucin glycoprotein, modified with tetrazine or norbornene, that quickly formed covalent bonds through an inverse electron-demand Diels–Alder reaction [3,16,28]. To assess whether the QCR could sense differences in the mechanical properties of the resulting hydrogels, we tested the form of the muc-gels at two final mucin concentrations of 15 and 25 mg/mL.

**Figure 1:**
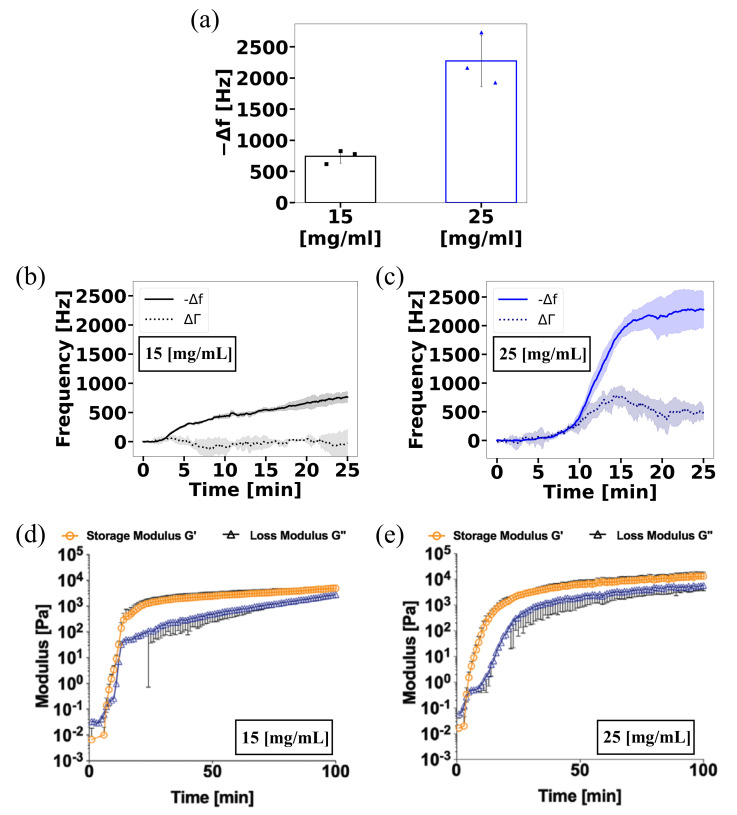
Characterisation of muc-gel formation. Using ViSQCT sensor @ 10MHz. (**a**) Comparison of Δf reached at the plateaulike stage after 25 min for 15 and 25 mg/mL muc-gels. (**b**) Time-dependent evaluation of Δf and ΔΓ for 15 mg/mL muc-gels. (**c**) Time-dependent evaluation of Δf and ΔΓ for 25 mg/mL muc-gels. The shaded area denotes the standard deviation as obtained from measurements of *n* = 3 independent samples. Rheological characterisation: (**d**) Time-dependent rheological evaluation for 15 mg/mL and (**e**) 25 mg/mL muc-gels. Rheological data were taken with permission from previously published work [3].

The measurements show a change in resonance frequency of the sensor approximately 5 min after mixing the two components (Figure 1a–c), irrespective of the concentrations. The resonance frequency of the sensor continued to drop until it reached a plateaulike state in approximately 25 min for higher concentration. As compared to a previously published plate-on-plate rotational rheology measurement on the same muc-gels (Figure 1d,e), some similarities appeared. Regarding gelation kinetics, both techniques suggested a slower increase in mechanical properties for the low-concentration muc-gels. In addition, both techniques suggested a slow down or plateau after 25 min of gelation. However, the rheological measurements show that only the storage modulus had reached a plateau after 25 min, whereas the loss modulus continued to increase for over an hour. This suggests that the QCR may be more sensitive to the elastic properties of the materials rather than to its viscous properties.

The frequency drop after 25 min of gelation was clearly high for the more concentrated muc-gel system. Differences in the rheological measurement were not as pronounced, with plateau moduli within the same order of magnitudes. This suggests differences in the mechanical properties sensed by both techniques or indicates that the low-concentration muc-gel on QCR did not finish gelating after 25 min. ΔΓ increased for the case of 25 mg/mL, reaching a plateau stage at about 20 min. This was due to the change in the viscoelastic properties of the material. However, for the 15 mg/mL concentration, ΔΓ did not show significant changes throughout the experiment. This may have been since, given the weak gelation, the viscosity change was not large enough to modify ΔΓ.

These results clearly demonstrate the potential to use QCR measurement to easily validate gelation events in covalently crosslinked hydrogel systems, but also to follow the gelling kinetics and assess differences in the mechanical properties of the gelled system due to polymer concentration.

In [27], the case of the bulk measurement of the hydrogel (taking into account the propagation of the shear wave from the QCR electrode to the fluid in contact with it) was illustrated. In this case, the response of the crystal could be described with the following equations:

(1) $$\Delta f=\frac{-f_{1}}{\pi Z_{q}}{\left(\left|G^{*}\right|\rho \right)}^{1/2}sin\left(\phi /2\right)$$

(2) $$\Delta \Gamma =\frac{f_{1}}{\pi Z_{q}}{\left(\left|G^{*}\right|\rho \right)}^{1/2}cos\left(\phi /2\right)$$

where $Z_{q}$ is the acoustic shear impedance (8.84 × $10^{6}$ kg/$m^{2}s$ for AT-cut quartz), ρ is the density, $\left|G^{*}\right|$ is the magnitude of the complex shear modulus, and ϕ is the viscoelastic phase angle of the medium.

**Figure 2:**
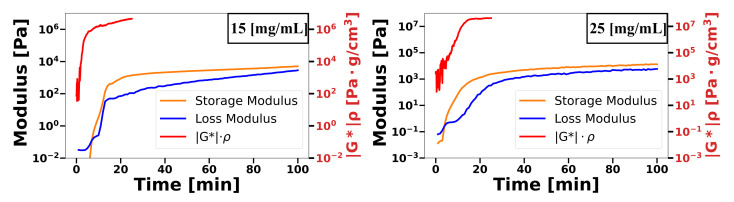
Comparison between rheological evaluation and complex modulus obtained from Δf and ΔΓ. (**left**) 15 mg/mL muc-gel; (**right**) 25 mg/mL muc-gel.

Figure 2 shows the modulus $\left|G^{*}\right|$ρ obtained from Δf and ΔΓ compared with G’ and G” obtained from the rheometric study. In both cases, the $\left|G^{*}\right|$ρ value had a larger amplitude than that of G’ and G”. This could be explained by considering that the obtained value was part of a product of two terms. On the one hand, the complex shear modulus, and on the other hand, the density. For the highest concentration, the curve was stabilised after 18 min; however, for the lowest concentration, no stabilisation was achieved, very similar to what was obtained in frequency. Spearman’s correlation coefficient was used to analyse the correlation between the data. A value greater than 0.9 was obtained between the data obtained by the sensor and the data measured by rheometry, which indicates a very strong relationship between the two.

### 2.2. QCR Can Measure the Gelation Kinetics of Physically Crosslinked Alginate Hydrogels

**Figure 3:**
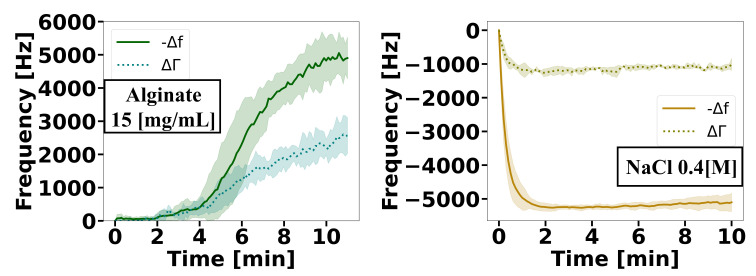
(**left**) Δf and ΔΓ changes after placing an alginate solution (15 mg/mL) on the sensor. Gelation was performed once the signal had stabilised. (**right**) Δf and ΔΓ change likely caused by a dilution effect following the exposure of the alginate to a solution of NaCl (0.4 M). The shaded area denotes the standard deviation as obtained from measurements of *n* = 3 independent samples.

We then tested whether QCR could also measure the gelling kinetics of alginate hydrogels crosslinked through divalent ions. Several differences with the covalently crosslinked mucin rendered this comparison interesting. First, Figure 3 shows the measurement of a 20 µL of alginate solution (1.5%, wt/v) loaded onto the QCR before exposure to calcium or barium and its gelation. The curve shows the gelation process until the frequency stabilised after approximately 10 min. Once this plateau had been reached, the gelation started with the addition of a small volume (4 µL) of ${\mathrm{BaCl}}_{2}$ or ${\mathrm{CaCl}}_{2}$ solutions. The addition of a similar volume of NaCl solution (0.4 M) to alginate caused a frequency increase (Figure 3), indicating that the samples were simply diluted by the addition of the solution and formed a less viscous fluid. Indeed sodium, which is used as a counterion to alginate, does not induce its gelation. When adding sodium to the alginate, the frequency also took approximately 2 min to reach equilibrium, so we consider this time to be a stabilisation period caused by factors such as damping produced when changing the physical properties of the sample.

**Figure 4:**
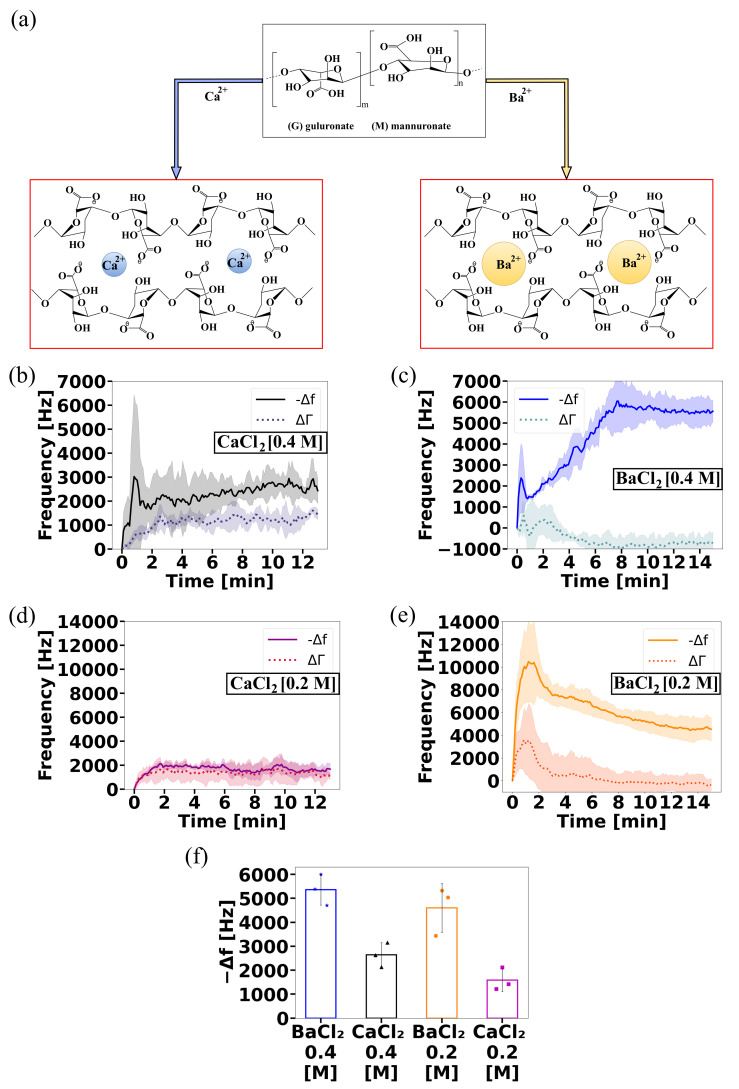
(**a**) Structure of alginate reacting with ${\mathrm{Ca}}^{2+}$ and ${\mathrm{Ba}}^{2+}$. Characterisation of alginate gel formation using two drivers with two concentrations using ViSQCT sensor @ 10 MHz. Time-dependent evaluation of Δf and ΔΓ: (**b**) ${\mathrm{CaCl}}_{2}$ (0.4 M). (**c**) ${\mathrm{BaCl}}_{2}$ (0.4 M). (**d**) ${\mathrm{CaCl}}_{2}$ (0.2 M). (**e**) ${\mathrm{BaCl}}_{2}$ (0.2 M). The shaded area denotes the standard deviation obtained from measurements of *n* = 3 independent samples. (**f**) Comparison of Δf plateaulike stage for the four cases.

Figure 4a illustrates the interaction of each type of ion with alginate. When adding the divalent ions on top of the alginate, the frequency of the QCR dropped, reflecting the increases in the viscous response of the material. The frequency shift values shown in Figure 4 were calculated from average frequency values from 12 min from adding barium ions and from 10 and 6 min after adding calcium at 0.4 and 0.2 M, respectively. The frequency drop was sensitive to two parameters that impact alginate gelation: the type of ions used and the molar ratio of divalent ions to alginate.

Barium ions led to a higher frequency drop than calcium did at both concentrations (Figure 4b–e). Decreasing the concentration of each ion by half also reduced the frequency drop. The concentration of the ionic crosslinker affects how many such crosslinking points are occupied on the alginate, which would directly affect the resulting gelling kinetics and mechanical properties. These results indicate that the QCR can indeed sense the ionic crosslinking of alginate into a hydrogel. Barium ions (0.195 nm) crosslink alginate into more stable gels than calcium does owing to them being larger than calcium ions (0.097 nm). Barium ions are a tighter fit within the alginate egg-box configuration that it adopts around such divalent ions [29]. Thus, stiffer gels can be obtained by crosslinking alginate solutions with barium ions compared to calcium ions at the same concentration.

There were also clear differences in the gelation kinetics of the gels. A sharp initial increase in frequency immediately after exposure to the divalent ions for ${\mathrm{BaCl}}_{2}$ and ${\mathrm{CaCl}}_{2}$ at the highest concentrations could have been the result of technical strain onto the crystal due to the sudden gelation of the alginate solution (Figure 4b,c). This was also observed for ${\mathrm{BaCl}}_{2}$ at the lowest concentration (Figure 4e), indicating that ${\mathrm{BaCl}}_{2}$ generated a strong and impulsive crosslinking with both concentrations. The lower concentration of ${\mathrm{CaCl}}_{2}$ did not induce this initial drop (Figure 4d), perhaps due to the lower crosslinking extent and strength compared to those in other conditions. To assess whether the initial sharp decrease in frequency could have been due to the development of the ionic crosslinking within the gel, we estimated the diffusion times of both ions. For ${\mathrm{CaCl}}_{2}$, considering a diffusion constant $D=0.79\times 10^{-5}$${\mathrm{cm}}^{2}/s$ [30] and a thickness = 0.502 mm, the diffusion time was 2.7 min. For ${\mathrm{BaCl}}_{2}$, maintaining the thickness and $D=0.84\times 10^{-5}$${\mathrm{cm}}^{2}/s$ [30], the diffusion time was 2.5 min. These diffusion times are within the timescale of these sharp frequency increases that took about 1 min to develop.

Following the initial drop in frequency, progressive evolution and then stabilisation were seen that could have been due to further diffusion of the ions into the gel, reorganisation, and perhaps relaxing stresses in the gel. QCRs are measured in the region closest to the crystal surface. In water, with a QCR with $f_{0}$ = 10 MHz, this distance (penetration depth) is 178 nm. It is, therefore, possible to consider a slower stabilisation time in this region.

The behaviour of ΔΓ showed that, for the two ${\mathrm{BaCl}}_{2}$ concentrations, ΔΓ had a slight increase and then dropped to stability at 8 min. In contrast, when using ${\mathrm{CaCl}}_{2}$, ΔΓ increased and reached stability at 3 min. The fact that ${\mathrm{BaCl}}_{2}$ ions form a stronger crosslinking helps in explaining the behaviour of ΔΓ, as its increase is related to the rise in viscoelasticity and the softening of the material, and its decrease with the stiffness of the material [31].

All presented data are from three independent experiments, with the standard deviation of frequency shift represented in a light colour.

## 3. Conclusions

The results show a difference of 1.5 kHz between the high- and lower-concentration muc-gels. In the case of the alginate hydrogels, the highest amplitude frequency drops were obtained when using barium (around 5.5 and 4.7 kHz for 0.4 and 0.2 M, respectively). For barium ions, given the strength of the ionic coordination, a strong pulse was observed at the beginning of the process. When using calcium ions, frequency drops of about 2.5 and 1.5 kHz were recorded for 0.4 and 0.02 M, respectively. The higher frequencies with barium ions were expected since they generate stiffer gels due to their tighter fit within the alginate. In this work, we demonstrate that a low-cost, portable QCR sensor could be used to follow the gelling process and compare the resulting mechanical properties of hydrogels. Using two hydrogels systems with covalent and physical ionic crosslinking, we demonstrated the versatility of the approach. Although we did not quantitatively validate how these measurements compared with plate-on-plate rheology, we demonstrated how important qualitative features could be studied with such a technique. The kinetics of the frequency drop and the frequency drop at equilibrium can be used to compare hydrogel systems in their gelation kinetics, gelation mechanism, and provide an indication about their final mechanical properties. The device is easy to use and low-cost, allowing for any laboratory to access and implement it. When developing new crosslinking chemistry, such a sensor could be used to quickly assess whether crosslinking had occurred and compare strategies. The findings of this work show that a comparison between this type of characterisation and that carried out by rheology is possible. QCR could become an alternative technique by correlating the measured parameters with elastic and viscous moduli. Overall, this work provides the proof of concept for this class of sensors to be used as hydrogel formation monitors. Its low cost and compactness could allow for it to be included in high-throughput approaches, and further developing and improving this kind of sensor is worthwhile.

## 4. Materials and Methods

### 4.1. Covalently Crosslinked Mucin Hydrogels

**Figure 5:**
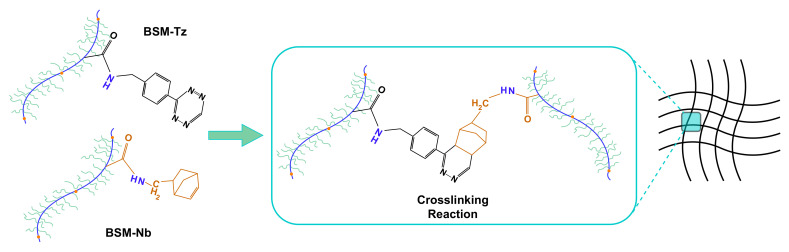
Tetrazine (Tz) and norbornene (Nb) conjugated to BSM and the crosslinking reaction [28].

Tetrazine (Tz) and norbornene (Nb) functionalities were introduced onto the bovine submaxillary mucin (BSM) molecules, following a previously published protocol [28] and resulting in BSM-Tz and BSM-Nb derivatives. When solutions of BSM-Tz and BSM-Nb are mixed together, they quickly form covalent bonds and form a hydrogel material (Figure 5). Such hydrogels (muc-gels) have interesting immune-modulating properties for biomedical applications.

The synthesis was performed as follows. MES buffer (0.1 M MES, 0.3 M NaCl, and pH 6.5) was used to predissolve BSM at 10 mg/mL. To this reaction mixture, 1-ethyl-3-(3-dimethylaminopropyl) carbodiimide (EDC; 4 mmol per gram of dry mucin) and N-hydroxysuccinimide (NHS; 4 mmol per gram of dry mucin) were added and stirred for 15 min. After that, 1 mmol of Tz and 2 mmol of Nb were added individually to generate BSM-Tz or BSM-Nb. The reaction mixtures were stirred overnight at 4 °C, and then dialysed for 2 days (100 kDa cutoff) against 300 $\times \;10^{-3}$ M NaCl and then MilliQ (MQ) H_2_O for 2 days. The samples were freeze-dried and stored at −20 °C. The schematic diagram of the chemical synthesis reaction of hydrogels and the reaction mechanisms are shown in Appendix A.

BSM-Tz and BSM-Nb were dissolved in PBS at 15 and 25 mg/mL. Then, a volume of 15 µL of each component was mixed (30 µL in total) then added immediately on the crystal surface to start the measurement.

### 4.2. Physically Crosslinked Alginate Hydrogels

**Figure 6:**
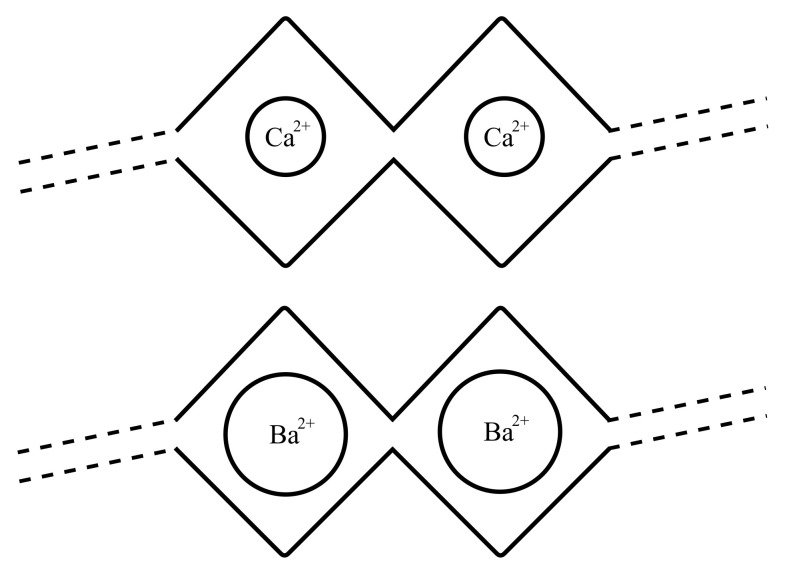
Egg-box model that illustrates the sizes of Ca^2+^ and Ba^2+^ ions that formed an ionic coordination with alginate.

The alginate polysaccharide was selected as an alternative hydrogel system to study. As opposed to the BSM–Tz/BSM–Nb system, alginate polymers can be ionically crosslinked by divalent ions (Figure 6), which present different kinetic and bond strengths compared to covalent bond formation [28,32]. With this system, we varied the strength of the crosslinking by selecting two crosslinking ions. Calcium, which was extensively used to crosslinked alginate into hydrogels, and barium that, owing to its larger size, results in more stable hydrogels, exhibiting less swelling.

Alginate (PRONOVA SLG20, NovaMatrix) was stirred overnight at 15 mg/mL using a saline solution (154 mM NaCl). ${\mathrm{BaCl}}_{2}$ and ${\mathrm{CaCl}}_{2}$ were also diluted at 0.4 and 0.2 M with NaCl. In the experiments, 20 µL of alginate was deposited on the crystal, causing it to spread over the entire surface. Then, the resonance frequency of the crystal was measured until it remained at a stable value. Once a stable frequency had been reached, a drop (4 µL) of ${\mathrm{BaCl}}_{2}$ (or ${\mathrm{CaCl}}_{2}$) was placed on the alginate to start the gelation process.

For both cases, gel formation was considered to be complete when the resonance frequency of the crystal showed a plateaulike state.

### 4.3. QCR Sensor

**Figure 7:**
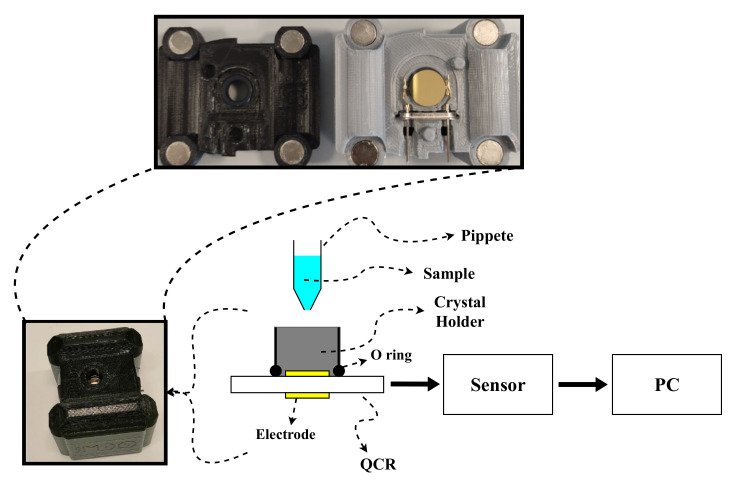
Experimental setup to measure hydrogel gelation process.

The ViSQCT project of the Bioinstrumentation and Nanomedicine Laboratory (LBN) of the Universidad Politécnica de Madrid (UPM) developed a portable and low-cost sensor based on QCRs (Figure 7) to measure the viscosity of a fluid with a small sample volume (tens of µL) [25,33]. As mentioned above, the most common use of QCRs is as QCMs, whose basis of operation was first established by Sauerbrey’s investigations, which describe the relationship between crystal resonance frequency shift and the added mass [34]. Subsequently, the connection between the frequency shift and the density–viscosity product of the fluid in contact with the crystal was given by the Kanazawa relationship [35].

The development of the sensor stemmed from using QCRs to monitor physical changes on the surface of the crystal. For this purpose, the methodology consists of measuring the resonance frequency and observing its variation when a fluid is in contact with the crystal.

Resonance frequency (*f*) obtention is achieved by exciting the crystal with a frequency sweep near the fundamental resonance frequency ($f_{0}$) and obtaining the electrical conductance (G) at each frequency. Then, the resonance frequency is located at the maximal conductance point. Frequency shift (Δf) and the shift in half-bandwidth at half-maximum (ΔΓ) are obtained and monitored. The bandwidth (Γ) change is related to the energy transferred from the crystal to the sample over time, and can provide information on the viscoelastic properties of the sample. The relationship between dissipation (D) and Γ is: D = 2Γ/*f* [31].

### 4.4. Experimental Setup

The experimental setup is illustrated in Figure 7. The QCR was placed inside a 3D-printed holder cell, where the liquid sample was dropped by pipette and allowed for the static measurement of the liquid. After the fluid addition, the holder was covered with parafilm to avoid the evaporation of the sample. Experiments were performed at room temperature. The QCR was connected to the sensor to measure the resonance frequency, and it was controlled by a LabVIEW (National Instruments) program developed in the LBN for this specific sensor. Lastly, the data were stored and analysed.

AT-cut quartz crystals with $f_{0}$ = 10 MHz, gold electrodes, electrode dimensions of 5 and 11 mm, roughness < 1 nm, and mounted onto an HC-51 holder were used (Figure 7). The crystals were purchased from Krystaly (Hradec Králové, Czech Republic). After each experiment, the crystal was cleaned using a 2% solution of sodium dodecyl sulfate, and immersed in a mixture of 10 mL of water, 2 mL of ammonia, and 2 mL of hydrogen peroxide for 10 min at 70 °C. Lastly, it was rinsed with distilled water and dried with air.

## Figures and Tables

**Figure A1:**
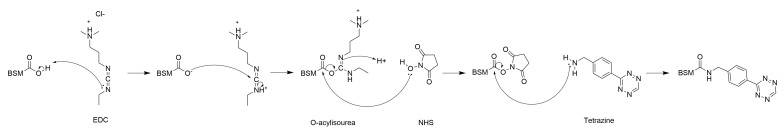
EDC NHS coupling (tetrazine).

**Figure A2:**
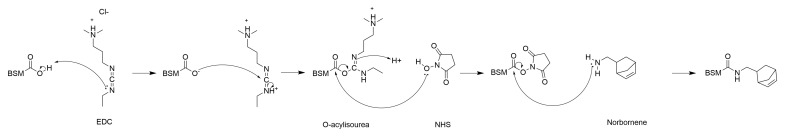
EDC NHS coupling (norbornene).

**Figure A3:**
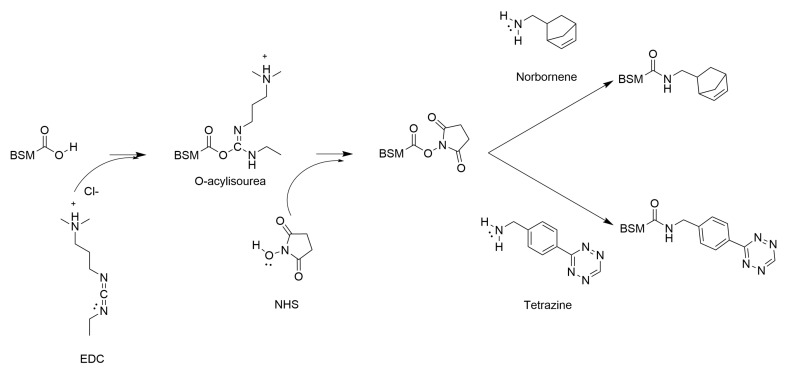
EDC NHS coupling (simplified).

**Figure A4:**
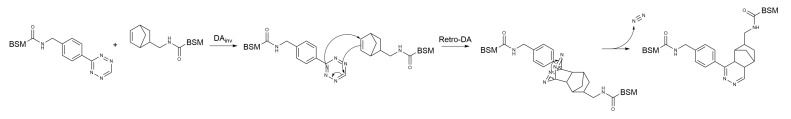
Reaction mechanism Inverse electron demand Diels–Alder.

## Data Availability

The data presented in this study are available on request from the corresponding author.

## Appendix A

EDC enables to increase the reactivity of carboxylic acid group present on BSM by converting it into an O-acylisourea intermediate. The addition of NHS stabilises the amine-reactive intermediate by forming an NHS ester. Then, an amine (in this case, 5-norbornene-2-methylamine or (4-(1,2,4,5-tetrazin-3-yl)phenyl)methanamine)) can react by an addition to the ester followed by the elimination of NHS.

Thanks to its low electron density, the tetrazine ring grafted onto BSM can react with the norbornene alkene function grafted onto another BSM in a cycloaddition (4 + 2). This reaction is the inverse electron-demand Diels–Alder reaction. The use of this click chemistry allows for the preparation of a BSM gel under mild conditions.
